# A Mechanism Exploration of Metabolic Syndrome Causing Nodular Thyroid Disease

**DOI:** 10.1155/2019/9376768

**Published:** 2019-11-26

**Authors:** Zexin Li, Lili Zhang, Yingshi Huang, Peixuan Yang, Wencan Xu

**Affiliations:** ^1^Health Care Center, The First Affiliated Hospital of Shantou University Medical College, Shantou 515041, China; ^2^Department of Endocrinology, The First Affiliated Hospital of Shantou University Medical College, Shantou 515041, China

## Abstract

**Background:**

Metabolic syndrome (MS) and its components have been demonstrated to facilitate the prevalence of thyroid nodules (TNs). The underlying pathogenesis needs to be elucidated.

**Methods:**

A total of 2722 subjects, who underwent health checkup in our institute from December 2014 to November 2018, were retrospectively and randomly collected. After exclusion, 2068 subjects were chosen, and their anthropic and clinical data were collected.

**Results:**

After matching age, gender, uric acid (UA), and creatinine (Cr) by propensity score matching (PSM), subjects with MS had higher prevalence of TNs than those without MS, as well as higher thyroid-stimulating hormone (TSH) and inflammatory levels, indicated by the higher white blood cell (WBC), lymphocyte (LY), and monocyte/high-density lipoprotein (Mo/HDL). After matching age, gender, UA, Cr, TSH, free triiodothyronine (FT3), thyroxine (FT4), WBC, NE, LY, Mo, NE/LY, LY/Mo, and Mo/HDL by PSM, no significant difference of the prevalence of TNs was found between MS and non-MS groups. Step logistic regression suggested glucose intolerance (GI), among all the components of MS, was an independent impact factor of TNs and was considered to contribute most to the formation of TNs. The prevalence of TNs was higher in the GI group after matching age, gender, body mass index (BMI), systolic blood pressure (SBP), diastolic blood pressure (DBP), fasting blood sugar (FBS), UA, Cr, triglyceride (TG), cholesterol (CHOL), HDL, and low-density lipoprotein (LDL).

**Conclusions:**

Patients with MS have a higher prevalence of TNs, probably due to the elevated TSH and inflammatory levels *in vivo*. Among the components of MS, glucose intolerance contributes most to the development of TNs.

## 1. Introduction

Thyroid nodules (TNs) are common presenting diseases for the clinical practitioner. TNs are usually detected incidentally by various imaging scanners performed for reasons beyond thyroid diseases or in a health checkup [1]. The prevalence of TNs is from 4% to 7% when estimated by palpation, convenient access by a practitioner [2]. While using more sensitive examination access, such as ultrasound, 13% to 67% subjects who undergo a routine health checkup are identified with TNs [3]. Females are more predisposed to TNs than males, and the prevalence of TNs increases with age [4]. Due to the high morbidity of TNs, studies focusing on risk factors of this disease are needed.

The association between TNs and metabolic syndrome (MS) is brought into focus in recent years. A case-control study, comprising 278 patients with MS and 261 controls, in a mild-to-moderate iodine-deficient region showed that patients with MS have a significantly higher prevalence of TNs, as well as the mean thyroid volume. All components of MS were found to be independent impact factors for the enlargement of thyroid volume. Patients with insulin resistance, coexisting and interacting with MS, had an odds ratio of 3.2 for the presence of TNs, suggesting a positive correlation between insulin resistance and the formation of TNs [5]. A cross-sectional study based on a Chinese community in a moderate iodine intake area found that the prevalence of TNs is significantly higher in individuals with MS than in those without MS. Logistic regression indicates that some components of MS, such as waist circumference, fasting plasma glucose, and hypertension, are positively associated with TN formation [6]. In a prospective cohort study in an iodine-adequate area, individuals with greater waist circumference have a higher probability of developing TNs after 3-year follow-up. The elevated blood triglyceride is a risk factor for the formation of new TNs [7].

Although MS and its components have been demonstrated to facilitate the prevalence of TNs, the underlying pathogenesis needs to be elucidated. This research hypothesized the increased prevalence of TNs in MS patients depended on the elevated TSH and inflammatory levels coexisting with MS, which would be confirmed by a clinical study.

## 2. Materials and Methods

### 2.1. Study Design and Subjects

A clinical cohort was used to investigate the association between TNs and MS. A total of 2722 subjects, who underwent health checkup in our department in the First Affiliated Hospital of Shantou University Medical College from December 2014 to November 2018, were retrospectively and randomly collected. All the subjects had comprehensive and full checkups, including routine physical examination, laboratory examinations, and thyroid ultrasound. Exclusion criteria included the following: a thyroid surgery history found by ultrasound; autoimmune thyroiditis and Graves' disease detected by a combination of ultrasound and thyroid function; other diseases with thyroid dysfunction; severe diseases of the heart, lung, liver, and kidney; and apparent infection identified by the conclusion of the checkup reports. Thyroid dysfunction was defined as serum thyroid-stimulating hormone (TSH), free thyroxine (free T4, FT4), or free triiodothyronine (free T3, FT3), which was out of the reference range of 5.5–0.35 mIU/L, 22.7–11.5 pmol/L, or 6.5–3.5 pmol/L, respectively. After excluding 654 ineligible subjects, 2068 eligible subjects were enrolled in this study. The collected data included age, gender, metabolic parameters, thyroid function, inflammatory indicators mainly from the blood routine test, and presence or absence of TNs based on ultrasound. TN disease was defined as any nodular lesion that is different from the normal parenchyma of the thyroid gland based on ultrasound in this study [1]. Metabolic parameters included body mass index (BMI), systolic and diastolic blood pressure (SBP and DBP), fasting blood sugar (FBS), serum uric acid (UA), creatinine (Cr), triglyceride (TG), cholesterol (CHOL), low-density lipoprotein (LDL), and high-density lipoprotein (HDL). Inflammatory indicators included white blood cell (WBC), neutrophil (NE), lymphocyte (LY), monocyte (Mo), NE/LY ratio, LY/Mo ratio, and Mo/HDL ratio. The MS definition by the Chinese Diabetes Society (2004) was having ≥3 components, which are listed as follows: overweight or obesity: BMI ≥ 25 kg/m^2^; dyslipidemia: triglycerides ≥1.7 mmol/L and/or fasting HDL cholesterol <0.9 mmol/L in male or <1.0 mmol/L in female; hypertension: blood pressure ≥140/90 mmHg and/or medication; and glucose intolerance: fasting plasma glucose ≥6.1 mmol/L and/or medication [8]. This study was approved by the Ethics Committee of the First Affiliated Hospital of Shantou University Medical College and in accordance with the 1964 Helsinki declaration and its later amendments or comparable ethical standards.

### 2.2. Statistical Analysis

R software (v3.5.3) was used for statistical analysis of anthropic and clinical data. Continuous data and dichotomous data were given as mean (S.D.) and number (%), respectively. Normal distribution of the continuous data was estimated by Q-Q plot. Propensity score matching (PSM) with nearest-neighbor matching (caliper = 0.10, ratio = 2) was performed to correct the potential confounders. Standardized mean difference (SMD) was calculated to assess the differences of the variables between groups and whether the PSM reduced the differences after matching. Continuous data between groups were compared by the *t*-test or Wilcoxon test according to the distribution of the data. Stepwise logistic regression (direction = “both”) was used to investigate the association between the components of MS and TNs. *P* < 0.05 was considered as statistically significant.

## 3. Results

### 3.1. Anthropic and Clinical Characteristics of All Subjects

A total of 2068 eligible subjects were enrolled and assigned into two groups, MS group and non-MS group. The baseline of the characteristics of all subjects is shown in Table 1. As shown in Table 1, subjects with MS have higher age, BMI, SBP, DBP, FBS, UA, Cr, TG, CHOL, LDL, WBC, NE, LY, Mo, Mo/HDL, and TNs, but lower female proportion and HDL.

### 3.2. Association between MS and TNs

PSM was used to account for the confounders. After matching age, gender, UA, and Cr, 118 subjects with MS, reflected by the higher BMI, SBP, DBP, FBS, TG, and lower HDL, were matched to 231 subjects without MS. The baseline of age, gender, UA, and Cr had no significant differences between the two groups. The MS group had higher TSH and inflammatory levels, indicated by the higher WBC, LY, and Mo/HDL. Notably, the prevalence of TNs was higher in the MS group than in the non-MS group (61.9% vs. 46.8%, *P*=0.01; Table 2). To investigate whether MS could affect the prevalence of TNs independently, we matched age, gender, UA, Cr, TSH, FT3, FT4, WBC, NE, LY, Mo, NE/LY, LY/Mo, and Mo/HDL by PSM. One hundred fourteen subjects with MS were matched to 219 subjects without MS. There were no significant differences in all the matched variables between the two groups. Likewise, there was no significant difference in the prevalence of TNs between the MS and non-MS groups (62.3% vs. 58.0%, *P*=0.523; Table 3). This finding indicates that the effect of MS on the development of TNs is TSH- and inflammation-dependent.

### 3.3. Association between the Components of MS and TNs

Step logistic regression was used to investigate the association between the components of MS and TNs. Among all the components of MS, glucose intolerance (GI) was an independent impact factor of TNs (OR: 1.501, 95% CI: 1.065–2.131; Table 4) and was considered to contribute most to the formation of TNs. To verify this finding, we stratified all the subjects into two groups, GI group (224 subjects) and non-GI group (1844 subjects). After matching age, gender, BMI, SBP, DBP, FBS, UA, Cr, TG, CHOL, HDL, and LDL, 212 subjects with GI were matched to 385 subjects without GI. No significant differences in the matched variables were found between the two groups. As we expected, the prevalence of TNs was higher in the GI group, as well as two inflammatory parameters, NE and NE/LY (Table 5). Thus, GI might contribute most to MS promoting TNs formation, probably due to the elevated inflammatory levels related to GI.

## 4. Discussion

The association between MS and TNs has been demonstrated by different types of studies, such as the case-control, cross-sectional, and prospective cohort studies. This study also confirmed a positive association between MS and TNs. Patients with MS had a higher risk to suffer TN disease, even after adjusting age and gender. It is still unknown whether MS stimulates the growth and proliferation of thyroid cells directly to form a nodule, or in an indirect way, by some active biochemicals *in vivo* coexisting with MS. To investigate this, we further matched the parameters of thyroid function and inflammation and found that the presence of MS alone did not have an effect on the prevalence of TNs. This may suggest that MS is not an independent risk factor of TNs, rather than relying on the abnormity of thyroid function and the elevated inflammatory levels.

The association between TNs and TSH is controversial. A positive relationship is found in the old persons, including 590 men and 597 women aged from 65 to 88 years old, in a population-based study, in which the subjects were stratified according to the quantiles of the serum TSH. Subjects in the top quartile have a significantly higher prevalence of MS than those in the bottom quartile (OR = 1.68, 95% CI: 1.19–2.37). After adjusting the confounders, including age, gender, alcohol consumption, total physical exercise, and smoking, the OR is still high (OR = 1.62, 95% CI: 1.15–2.32) [9]. Another study explored the association between TSH and MS in 1333 euthyroid German subjects. Subjects with a normal range of TSH were divided into two groups by median. In the upper TSH group, there are more persons with obesity, high triglycerides, and MS. In contrast, a lower TSH is related to a favorable metabolic status [10]. However, the absence of a positive relationship is also found in some studies. In obese and overweight African Americans, there is no significant association between serum TSH levels and MS. After correcting for age, gender, ethnicity, educational background, socioeconomic status, and smoking, no significant associations between TSH and the components of MS were found [11]. This negative result is consistent with that found in euthyroid Taiwanese individuals [12]. The controversial results probably are attributed to the slightly different definition of MS, adjusted confounders, and ethnicity- and disease-based population. In our study, we also found a positive relation between MS and TSH. After matching age and gender, subjects with MS had higher serum TSH levels compared to those without MS.

As we know, elevated TSH is the main cause of goiter and nodularity [13]. Several mechanisms have been detected. TSH binds and activates the TSH receptor and then induces production of intracellular cyclic AMP (cAMP). The increased cAMP activates protein kinase A (PKA) through a cAMP-dependent approach, and PKA subsequently phosphorylates nuclear factor cAMP-response element binding protein (CREB). CREB then activates the transcription of its targeted genes, which were responsible for thyroid cell proliferation [14]. TSH also can stimulate thyroid cell proliferation by inhibiting the expression of gene *SMAD3*, a member of the transforming growth factor-*β* (TGF-*β*) pathway that inhibits thyroid follicular cell proliferation [15]. Another regulation mechanism of TSH depends on the p70S6K-mediated effects on the localization of p27, by which TSH increases the number of cycling thyroid cells and therefore facilitates thyroid cell proliferation [16]. Hence, the elevated TSH, which tends to co-occur with MS, is a key link between MS and TNs.

The interaction between MS and inflammation has been investigated in depth in recent years. Inflammation plays a key role in the onset and progression of metabolic disorders. Some inflammatory markers, such as C-reactive protein, complement component 3, leukocyte, neutrophil, and lymphocyte, are found to increase in patients with MS [17, 18]. The development of MS is a chronic process, usually evolving from the overnutrition. The excessive energy will cause metabolic stress when it exceeds the storage capacity of metabolism-related organisms, such as adipose and muscular tissue. The metabolic stress will activate the inflammatory signaling, which increases the production of cytokines and causes low-grade inflammation *in vivo*, leading to the development of insulin resistance, a major pathogenesis of MS. Besides, the components of MS hyperglycemia and hyperlipidemia can activate inflammatory signaling by toll-like receptors, leading to a mild inflammatory response and insulin resistance [19]. In our study, subjects with MS have higher inflammatory levels, indicated by the higher WBC, NE, LY, and Mo/HDL, which supports the previous studies as well.

Chronic, low-grade inflammation is likely a risk factor of TNs. Euthyroid subjects infected with *Helicobacter pylori* (HP), a chronic infection in the stomach and a main cause of the gastric ulcer, have significantly increased risk of TNs, due to the elevated inflammatory levels caused by HP infection [20]. Another chronic infection by hepatitis C virus (HCV) is reported to take responsibility for an increased prevalence of malignant TNs, like papillary thyroid cancer. It is attributed to the inflammatory circumstance in the thyroid gland resulted from a range of inflammatory process induced by HCV infection [21]. Thus, inflammation, especially chronic, low-grade inflammation, plays a vital role in MS, promoting TN formation.

In this study, among the components of MS, glucose intolerance (GI) was determined to contribute most to the formation of TNs by step logistic regression. In the GI group, we also found a higher prevalence of TNs. This finding is similar to a previous study, in which insulin resistance and diabetes, instead of other components of MS, are all independent risk factors for TNs [22]. However, some studies present different results. One of the studies found that systolic blood pressure exceeds other components in contribution to TN formation [23]. Another study found obesity has a higher odds ratio for TNs than other components [24]. The different results are acceptable based on the different population and suggest a collective and accumulative effect of all the components of MS on TN development. Due to the elevated inflammatory levels found in the subjects with GI, which have been reported in a large number of studies [25], it is inferred that the effect of GI on TN formation partly relies on the elevated inflammatory levels in the body.

## 5. Conclusions

Patients with MS have a higher prevalence of TNs, probably due to the elevated TSH and inflammatory levels *in vivo*. Among the components of MS, glucose intolerance contributes most to the development of TNs. For the prevention of TN disease, management of patients with MS should take TSH and inflammation into account.

## Figures and Tables

**Table 1:** Anthropic and clinical characteristics of subjects with or without MS.

Variables	MS (*n* = 120)	Non-MS (*n* = 1948)	*P*	SMD
Age (years)	52.55 (13.65)	41.18 (13.39)	<0.001	0.841
Gender (female, *n*)	21 (17.5)	700 (35.9)	<0.001	0.426
Body mass index (kg/m^2^)	27.32 (2.56)	23.55 (5.00)	<0.001	0.951
Systolic blood pressure (mmHg)	140.76 (16.84)	119.36 (11.85)	<0.001	1.470
Diastolic blood pressure (mmHg)	92.95 (9.78)	77.67 (9.09)	<0.001	1.618
Fasting blood sugar (mmol/L)	6.57 (1.79)	5.33 (0.95)	<0.001	0.859
Uric acid (mmol/L)	461.68 (112.29)	393.13 (95.72)	<0.001	0.657
Creatinine (mmol/L)	80.08 (17.99)	75.90 (20.15)	0.012	0.219
Triglyceride (mmol/L)	2.40 (1.34)	1.30 (0.89)	<0.001	0.970
Cholesterol (mmol/L)	5.43 (0.94)	4.87 (0.88)	<0.001	0.614
High-density lipoprotein (mmol/L)	1.17 (0.26)	1.42 (0.33)	<0.001	0.822
Low-density lipoprotein (mmol/L)	3.20 (0.82)	2.87 (0.75)	<0.001	0.419
Thyroid-stimulating hormone (mIU/L)	2.00 (1.01)	1.84 (0.93)	0.060	0.170
Free triiodothyronine (nmol/L)	5.20 (0.52)	5.18 (0.55)	0.686	0.039
Free thyroxine (nmol/L)	15.85 (2.09)	16.01 (2.21)	0.440	0.074
White blood cell (10^9^ cells/L)	7.10 (2.11)	6.40 (1.60)	<0.001	0.376
Neutrophil (10^9^ cells/L)	3.89 (1.52)	3.57 (1.20)	0.005	0.236
Lymphocyte (10^9^ cells/L)	2.57 (0.79)	2.25 (0.61)	<0.001	0.452
Monocyte (10^9^ cells/L)	0.43 (0.15)	0.39 (0.13)	0.001	0.273
Neutrophil/lymphocyte ratio	1.60 (0.63)	1.67 (0.69)	0.225	0.110
Lymphocyte/monocyte ratio	6.43 (2.27)	6.14 (2.08)	0.217	0.132
Monocyte/high-density lipoprotein ratio	0.39 (0.18)	0.30 (0.13)	<0.001	0.583
Thyroid nodules (*n*)	75 (62.5)	911 (46.8)	0.001	0.320

Data were presented as mean (S.D.) or number (%). MS: metabolic syndrome; SMD: standardized mean difference.

**Table 2:** Anthropic and clinical characteristics of subjects with or without MS based on propensity score matching.

Variables	MS (*n* = 118)	Non-MS (*n* = 231)	*P*	SMD
Age (years)	51.96 (12.97)	50.03 (14.10)	0.215	0.143
Gender (female, *n*)	21 (17.8)	37 (16.0)	0.787	0.047
Body mass index (kg/m^2^)	27.36 (2.57)	23.78 (2.99)	<0.001	1.282
Systolic blood pressure (mmHg)	140.64 (16.95)	122.05 (14.65)	<0.001	1.173
Diastolic blood pressure (mmHg)	93.08 (9.81)	79.47 (10.97)	<0.001	1.307
Fasting blood sugar (mmol/L)	6.56 (1.80)	5.50 (1.01)	<0.001	0.727
Uric acid (mmol/L)	459.25 (111.30)	454.59 (104.83)	0.700	0.043
Creatinine (mmol/L)	79.87 (18.07)	81.56 (17.28)	0.397	0.095
Triglyceride (mmol/L)	2.41 (1.34)	1.51 (1.03)	<0.001	0.752
Cholesterol (mmol/L)	5.46 (0.91)	5.06 (0.90)	<0.001	0.440
High-density lipoprotein (mmol/L)	1.18 (0.26)	1.33 (0.32)	<0.001	0.513
Low-density lipoprotein (mmol/L)	3.22 (0.81)	3.05 (0.77)	0.061	0.211
Thyroid-stimulating hormone (mIU/L)	2.00 (1.02)	1.76 (0.85)	0.020	0.258
Free triiodothyronine (nmol/L)	5.21 (0.52)	5.26 (0.54)	0.398	0.096
Free thyroxine (nmol/L)	15.84 (2.10)	15.88 (2.14)	0.883	0.017
White blood cell (10^9^ cells/L)	7.11 (2.12)	6.63 (1.80)	0.026	0.246
Neutrophil (10^9^ cells/L)	3.91 (1.53)	3.68 (1.32)	0.138	0.164
Lymphocyte (10^9^ cells/L)	2.57 (0.79)	2.33 (0.65)	0.003	0.326
Monocyte (10^9^ cells/L)	0.43 (0.15)	0.42 (0.14)	0.516	0.072
Neutrophil/lymphocyte ratio	1.61 (0.63)	1.65 (0.61)	0.529	0.071
Lymphocyte/monocyte ratio	6.44 (2.29)	6.08 (2.83)	0.230	0.141
Monocyte/high-density lipoprotein ratio	0.38 (0.18)	0.34 (0.15)	0.008	0.293
Thyroid nodules (*n*)	73 (61.9)	108 (46.8)	0.010	0.307

Matching variables included age, gender, uric acid, and creatinine. Data were presented as mean (S.D.) or number (%). MS: metabolic syndrome; SMD: standardized mean difference.

**Table 3:** Association between MS and TNs based on propensity score matching.

Variables	MS (*n* = 114)	Non-MS (*n* = 219)	*P*	SMD
Age (years)	51.96 (12.97)	49.84 (14.20)	0.177	0.027
Gender (female, *n*)	21 (17.8)	41 (17.7)	1.000	0.016
Body mass index (kg/m^2^)	27.36 (2.57)	23.51 (2.43)	<0.001	1.281
Systolic blood pressure (mmHg)	140.48 (16.82)	123.68 (12.06)	<0.001	1.148
Diastolic blood pressure (mmHg)	93.04 (9.73)	80.60 (8.55)	<0.001	1.359
Fasting blood sugar (mmol/L)	6.58 (1.83)	5.71 (1.80)	<0.001	0.478
Uric acid (mmol/L)	455.29 (109.43)	446.34 (104.02)	0.465	0.084
Creatinine (mmol/L)	80.11 (18.19)	77.88 (16.68)	0.263	0.128
Triglyceride (mmol/L)	2.39 (1.36)	1.69 (1.27)	<0.001	0.538
Cholesterol (mmol/L)	5.44 (0.91)	5.24 (0.95)	0.062	0.218
High-density lipoprotein (mmol/L)	1.18 (0.26)	1.27 (0.31)	0.010	0.309
Low-density lipoprotein (mmol/L)	3.21 (0.81)	3.20 (0.75)	0.910	0.013
Thyroid-stimulating hormone (mIU/L)	2.00 (1.03)	1.97 (0.98)	0.745	0.037
Free triiodothyronine (nmol/L)	5.21 (0.52)	5.22 (0.53)	0.783	0.032
Free thyroxine (nmol/L)	15.88 (2.09)	15.84 (1.99)	0.838	0.024
White blood cell (10^9^ cells/L)	7.09 (2.14)	7.10 (2.01)	0.973	0.004
Neutrophil (10^9^ cells/L)	3.92 (1.56)	3.92 (1.47)	1.000	0.000
Lymphocyte (10^9^ cells/L)	2.53 (0.76)	2.53 (0.73)	0.959	0.006
Monocyte (10^9^ cells/L)	0.43 (0.16)	0.43 (0.15)	0.816	0.027
Neutrophil/lymphocyte ratio	1.63 (0.63)	1.66 (0.92)	0.665	0.039
Lymphocyte/monocyte ratio	6.41 (2.31)	6.29 (1.96)	0.611	0.057
Monocyte/high-density lipoprotein ratio	0.38 (0.17)	0.37 (0.17)	0.558	0.067
Thyroid nodules (*n*)	71 (62.3)	127 (58.0)	0.523	0.088

Matching variables included age, gender, uric acid, creatinine, thyroid-stimulating hormone, free triiodothyronine, free thyroxine, white blood cell, neutrophil, lymphocyte, monocyte, neutrophil/lymphocyte ratio, lymphocyte/monocyte ratio, and monocyte/high-density lipoprotein ratio. MS: metabolic syndrome; TNs: thyroid nodules; SMD: standardized mean difference.

**Table 4:** Independent impact factors for TNs in the euthyroid population.

Variables	*β*	SE	Wald *χ*^2^	*P*	OR	95% CI of OR
Age	0.060	0.004	13.537	<0.001	1.062	[1.053, 1.071]
Gender	1.297	0.123	10.510	<0.001	3.659	[2.877, 4.669]
Glucose intolerance	0.406	0.177	2.299	0.021	1.501	[1.065, 2.131]
Uric acid	0.002	0.001	3.257	0.001	1.002	[1.001, 1.003]
Cholesterol	−0.269	0.125	−2.144	0.032	0.764	[0.596, 0.975]
Low-density lipoprotein	0.320	0.146	2.194	0.028	1.377	[1.037, 1.836]

TNs: thyroid nodules; *β*: partial regression coefficient; SE: standard error of partial regression coefficient; OR: odds ratio; CI: confidence interval. Stepwise logistic regression was performed. Dependent variable was thyroid nodules. Independent variables were age, gender, uric acid, creatinine, cholesterol, low-density lipoprotein, and the components of metabolic syndrome (obesity, hypertension, hypertriglyceridaemia, low high-density lipoprotein, and glucose intolerance).

**Table 5:** Association between GI and TNs based on propensity score matching.

Variables	GI (*n* = 212)	Non-GI (*n* = 385)	*P*	SMD
Age (years)	53.86 (13.10)	52.12 (12.32)	0.107	0.137
Gender (female, *n*)	76 (35.8)	120 (31.2)	0.283	0.099
Body mass index (kg/m^2^)	24.80 (2.77)	24.82 (9.72)	0.420	0.002
Systolic blood pressure (mmHg)	128.38 (15.62)	126.85 (14.55)	0.231	0.101
Diastolic blood pressure (mmHg)	83.65 (10.32)	82.86 (10.41)	0.372	0.077
Fasting blood sugar (mmol/L)	7.46 (2.07)	5.28 (0.39)	<0.001	1.465
Uric acid (mmol/L)	409.29 (100.13)	415.09 (96.72)	0.489	0.059
Creatinine (mmol/L)	77.78 (22.21)	78.70 (18.57)	0.228	0.045
Triglyceride (mmol/L)	1.67 (1.09)	1.72 (1.34)	0.898	0.037
Cholesterol (mmol/L)	5.34 (0.91)	5.34 (0.96)	0.976	0.003
High-density lipoprotein (mmol/L)	1.32 (0.33)	1.31 (0.31)	0.676	0.035
Low-density lipoprotein (mmol/L)	3.25 (0.78)	3.24 (0.79)	0.887	0.012
Thyroid-stimulating hormone (mIU/L)	1.83 (0.98)	1.89 (0.90)	0.499	0.057
Free triiodothyronine (nmol/L)	5.11 (0.53)	5.18 (0.55)	0.124	0.133
Free thyroxine (nmol/L)	15.93 (2.18)	15.61 (2.17)	0.087	0.147
White blood cell (10^9^ cells/L)	6.86 (1.94)	6.64 (1.73)	0.141	0.124
Neutrophil (10^9^ cells/L)	3.91 (1.49)	3.65 (1.22)	0.023	0.189
Lymphocyte (10^9^ cells/L)	2.34 (0.70)	2.37 (0.67)	0.574	0.048
Monocyte (10^9^ cells/L)	0.41 (0.14)	0.41 (0.14)	0.743	0.028
Neutrophil/lymphocyte ratio	1.78 (0.92)	1.62 (0.64)	0.026	0.210
Lymphocyte/monocyte ratio	6.14 (2.29)	6.14 (2.04)	0.971	0.003
Monocyte/high-density lipoprotein ratio	0.34 (0.16)	0.34 (0.16)	0.763	0.026
Thyroid nodules (*n*)	148 (69.8)	228 (59.2)	0.013	0.223

Matching variables included age, gender, body mass index, systolic blood pressure, diastolic blood pressure, uric acid, creatinine, triglyceride, cholesterol, high-density lipoprotein, and low-density lipoprotein. GI: glucose intolerance; TNs: thyroid nodules; SMD: standardized mean difference.

## Data Availability

The data used to support the findings of this study are available from the corresponding author upon request.

## Additional Points

*Limitation*. This study is a cross-sectional study. The causality between MS and TNs was inferred by this study in combination with other previous studies. Therefore, a prospective study is needed to demonstrate this causality in the future, which will be our next procedure.
